# A Comparative Transcriptome and WGCNA of Tomato Reveals Hub Genes and a Hormone-Mediated Defense Network Against *Ralstonia solanacearum*

**DOI:** 10.3390/biology15060509

**Published:** 2026-03-22

**Authors:** Chuying Yu, Xiaofang Wang, Chunchun Qin, Yi Liu, Guiyun Gan, Liangyu Cai, Rui Xiang, Yaqin Jiang, Weiliu Li, Qihong Yang, Yikui Wang

**Affiliations:** 1 Institute of Vegetable Research, Guangxi Academy of Agricultural Sciences, Nanning 530007, China; yuchuying@gxaas.net (C.Y.); wangxf@njau.edu.cn (X.W.); 17396706625@163.com (C.Q.); lumierelaw@webmail.hzau.edu.cn (Y.L.); ggyun19@163.com (G.G.); liangyuc5581@163.com (L.C.); xiangrui771@gmail.com (R.X.); jiangyaqin@gxaas.net (Y.J.); liweiliu@gxaas.net (W.L.); yangqihong@gxaas.net (Q.Y.); 2 College of Resources and Environmental Sciences, Nanjing Agricultural University, Nanjing 210095, China; 3 College of Agriculture, Guangxi University, Nanning 530004, China

**Keywords:** tomato, bacterial wilt-resistance, weighted gene co-expression analysis network, comparative transcriptome

## Abstract

Bacterial wilt caused by *Ralstonia solanacearum* is a major constraint on tomato (*Solanum lycopersicum* L.) production, yet the molecular basis of quantitative resistance remains poorly understood. In this study, comparative transcriptome profiling was performed on resistant (‘ZM3’) and susceptible (‘ZM86’) tomato inbred lines following pathogen inoculation in roots, stems, and leaves. Differential expression analysis and weighted gene co-expression network analysis (WGCNA) were conducted to identify resistance-associated regulatory modules and hub genes. The results revealed distinct gene expression patterns between the two genotypes after infection. Several co-expression modules were significantly associated with resistance or susceptibility traits. Functional enrichment analysis showed that differentially expressed genes were mainly involved in plant hormone signal transduction, plant–pathogen interaction, phenylpropanoid biosynthesis, and cell wall modification. Genes related to ethylene and salicylic acid signaling were strongly induced following infection, whereas brassinosteroid-associated genes showed genotype-dependent expression patterns. Network analysis further identified several hub genes within defense-related modules, including ACO (*Solyc04g007980*), ERF1 (*Solyc09g091950*), MAPK9, receptor-like kinase RLK (*Solyc07g006770*), and a dirigent family gene (*Solyc10g008900*). Taken together, our results suggest that tomato resistance to *Ralstonia solanacearum* involves a coordinated defense network integrating hormone-mediated transcriptional regulation and structural reinforcement, and provides candidate genes for breeding bacterial wilt-resistant cultivars.

## Simple Summary

Tomato is a globally important crop that is severely threatened by bacterial wilt caused by *Ralstonia solanacearum*. The disease can lead to substantial yield losses. We conducted transcriptome analysis of the root, stem, and leaf tissues from resistant and susceptible tomato lines to elucidate the molecular basis of resistance. The results revealed a multi-layered defense system coordinated by key hub genes. These genes regulate cell-wall reinforcement, hormone signaling, and immune activation, collectively restricting pathogen colonization. The identified genes offer practical targets for marker-assisted selection and genome editing for the development of wilt-resistant tomato varieties. This strategy supports sustainable agriculture by reducing dependence on chemical pesticides.

## 1. Introduction

Tomato (*Solanum lycopersicum*) is one of the most economically important vegetable crops worldwide, with a global cultivation area of approximately 5 million hectares and an annual production of approximately 182 million tons [1]. However, its yield and productivity are seriously threatened by bacterial wilt caused by the soil-borne pathogen *Ralstonia solanacearum*, which infects more than 250 plant species and results in substantial crop losses. *R. solanacearum* enters host plants via the roots, spreads through apoplastic intercellular spaces, and subsequently colonizes the xylem, where excessive bacterial proliferation blocks vascular flow, leading to plant wilting [2]. The control of bacterial wilt remains challenging owing to the high virulence of the pathogen and its ability to persist in the environment for extended periods [3]. Multiple approaches have been applied worldwide to control tomato diseases, including the use of resistant cultivars and cultural, physical, chemical, and biological measures [4], yet their effectiveness remains limited [5]; consequently, the identification of resistance-associated genes and the breeding of disease-resistant tomato varieties represent the most effective long-term strategies.

Bacterial wilt, a destructive pathogen, was first reported in potatoes, tomato, and eggplants in 1896 [6]. Subsequent studies have shown that the swimming motility of *R. solanacearum*, along with the production of cell wall–degrading enzymes, type III secretion system activity, and exopolysaccharide synthesis, are essential for host cell disruption, bacterial proliferation, and pathogenicity during infection [7,8,9]. Plants have developed sophisticated immune systems to perceive and counteract pathogen invasion, primarily through the coordinated regulation of defence-related genes by diverse transcription factors, including WRKY, bHLH, NAC, AP2/ERF, MYB, and bZIP. These regulators function synergistically within abscisic acid (ABA), salicylic acid (SA), jasmonic acid (JA), ethylene (ET), and reactive oxygen species (ROS)-mediated signaling networks [10]. For example, the transcription factor *SlWRKY75* enhances tomato resistance to bacterial wilt by activating antioxidant and defense enzyme activities, modulating the JA and SA signaling pathways, and maintaining reactive oxygen species homeostasis [10]. In Solanaceous crops, *SmDDA1b* enhances resistance via the *SmNAC*-mediated SA pathway [11], whereas Ca16R from pepper positively regulates resistance in *Nicotiana benthamiana* by promoting SA signaling and suppressing JA signaling [12]. Protein kinase signaling pathways (e.g., mitogen-activated protein kinases and MAPKs) play essential roles in plant disease resistance [13]. Members of the *SlCRLK1L* gene family in tomato contribute to defense responses against *Pseudomonas syringae* [14]. Currently, the tomato cultivar Hawaii 7996 (H7996) is regarded as the most effective source of resistance to diverse *R. solanacearum* strains [10]. Recent studies have shown that the previously resistant tomato cultivar Hawaii 7996 can be effectively infected with the highly virulent type II strain ES5-1 [15]. Therefore, identifying the key resistance factors and developing new disease-resistant varieties are both urgent and essential.

RNA sequencing (RNA-seq) is a direct, cost-effective method for the genome-wide identification of resistance-related genes [16]. Combined with weighted gene co-expression network analysis (WGCNA), this approach enables the detection of gene modules associated with specific traits, facilitating the identification of key candidate genes and metabolic pathways [17]. Integrated transcriptome analyses combined with WGCNA workflows have identified key modules and hub genes for disease resistance in diverse species; for example, in rice, modules such as saddlebrown, mediumorchid, and darkolivegreen helped delineate blast resistance mechanisms across infection stages [18]; in cotton, five resistance-specific modules were identified following *Verticillium dahlia* infection [19]; and in potato, hub transcription factors such as *WRKY33*, *MYB*, and *NAC* were identified within the lightsteelblue1 module [20]. Thus, we combined differential expression analysis (differentially expressed genes, DEGs) with WGCNA to identify the key genes related to resistance gene sets and regulatory networks in molecular breeding programs.

In this study, physiological indices were compared between resistant ‘ZM3’ and susceptible ‘ZM86’ tomato genotypes following inoculation with *R. solanacearum*. Comparative transcriptome analysis was conducted on the root, stem, and leaf tissues. Gene expression patterns associated with stress-related hormone pathways were systematically analyzed. Differentially expressed genes (DEGs) were subjected to Gene Ontology (GO) and Kyoto Encyclopedia of Genes and Genomes (KEGG) enrichment analyses. Using WGCNA, hub genes linked to *R. solanacearum* infection were identified, and modules highly correlated with bacterial treatment were characterized. Key nodal genes in the co-expression network were further investigated. Given that the genetic basis of quantitative resistance to *R. solanacearum* in tomato remains largely unresolved, we hypothesized that tomato resistance is mediated by coordinated hormone–immune signaling modules identifiable through transcriptome profiling and network analysis. Collectively, these results provide a theoretical basis for identifying resistance-related genes and advancing genetic engineering and molecular breeding strategies to improve bacterial wilt resistance in tomato.

## 2. Materials and Methods

### 2.1. Plant Materials, Growth Conditions, and Bacterial Inoculation

Two tomato inbred lines with contrasting responses to bacterial wilt were used in this study: ‘ZM3’ (R), a highly resistant line, and ‘ZM86’ (S), a susceptible line. These inbred lines were grown in trays with nutrient soil after germination and placed in a growth chamber under 20,000 lx light intensity, 75% relative humidity, 25 °C, and a 16 h/8 h light/dark photoperiod. After one week, seedlings with uniform growth were selected and transplanted into 7 × 7 cm pots containing nutrient-rich soil for further cultivation. When the plants reached the 4–5 true leaf stage, they were inoculated with *R. solanacearum* FJ91 using the root wounding-immersion method.

The bacterial suspension was adjusted to an OD_600_ = 0.1 (approx. 10^8^ CFU/mL) after activation. Lateral roots near the main root were wounded with a sterile blade, and 30 mL of the bacterial suspension was applied to each plant. The control plants received 30 mL of sterile water. Three technical replicates were established per genotype, with each replicate containing ≥ 15 plants in a randomized complete block design.

Inoculated plants were transferred to a climate-controlled room (30 °C, 80% RH, 12 h photoperiod). Root, stem, and leaf samples from both experimental and control groups were collected at 0 and 4 days post-inoculation (dpi). The 4 dpi time point was selected because clear disease symptoms began to appear in the susceptible genotype at this stage, indicating active pathogen infection and host responses. Three biological replicates per treatment group were flash-frozen in liquid nitrogen and stored at −80 °C.

### 2.2. RNA Sequencing and Transcriptome Data Processing

The Vezol Reagent (Vazyme, Nanjing, China) was used to extract total RNA from the samples. RNA purity was assessed using a NanoDrop 2000 spectrophotometer (Thermo Fisher Scientific, Waltham, MA, USA), and RNA integrity was verified using 1% agarose gel electrophoresis. Subsequently, strand-specific cDNA libraries were constructed by Shanghai Personal Biotechnology Co., Ltd. (Shanghai, China) using the Illumina TruSeq Standard Total RNA Library Prep Kit (Illumina, San Diego, CA, USA), and paired-end 150 bp sequencing was performed on the Illumina NovaSeq 600 platform. Raw reads were subjected to quality control using FastQC (v0.11.9). Low-quality bases and adapter sequences (Phred score < 20) were trimmed using Trimmomatic (v0.39) [21]. The resulting clean reads were aligned to the tomato reference genome SL4.0 (ITAG4.0, https://solgenomics.net/, accessed on 1 January 2026) using HISAT2 (v2.2.1) [22], and gene expression levels were quantified as transcripts per million (TPM) using StringTie (v2.2.1) [23].

### 2.3. Transcriptomic Differential Expression Genes Analysis

Differentially expressed genes (DEGs) were identified using the R package DESeq2 (v1.46.0) [24] with thresholds of |log2 fold change (FC)| > 1 and adjusted *p*-value (padj) < 0.05. Principal component analysis (PCA) was performed based on TPM values using the FactoMineR package (version 2.13) [25], and the results were visualized using factoextra (version 1.0.7) [26] and ggplot2 (version 3.4.4) [27]. The WGCNA package (version 1.72-1) [17] was used to calculate Pearson correlation coefficients and construct gene co-expression networks. Volcano plots were generated for visualization of DEGs, and Venn diagrams were produced using Jvenn [28]. GO [29] and KEGG [30] enrichment analyses of DEGs were conducted using TBtools II (v2.032) [31], and the top 20 most significant terms (*p* < 0.05) were retained.

### 2.4. qRT-PCR Validation of Key Genes

The primers were designed using ‘Primer3-py’ (v2.0.1, https://libnano.github.io/primer3-py/index.html, accessed on 3 January 2026) (Primers are in Table S2). cDNA was synthesized using a HiScript II 1st Strand cDNA Synthesis Kit (Vazyme, Nanjing, China). Quantitative real-time PCR (qRT-PCR) was performed using beta-Actin as the internal reference gene. The 20 µL qRT-PCR reaction mixture consisted of: 1 µL of cDNA, 1 µL each of the forward and reverse primers, 7 µL of ddH_2_O, and 10 µL of ChamQ Universal SYBR qPCR Master Mix (Vazyme, Nanjing, China). Each treatment included three biological and three technical replicates. Amplification was conducted on a Roche LightCycler 480 using the following protocol: 95 °C for 120 s; followed by 45 cycles of 95 °C for 5 s and 59 °C for 30 s. Relative gene expression was calculated using the 2^−ΔΔCt^ method [32], Prior to statistical analysis, expression values were log-transformed, and normality was assessed using the Shapiro–Wilk test. When the assumptions of normality and variance homogeneity were satisfied, Student’s *t*-test was applied; otherwise, Welch’s *t*-test was used. Statistical analyses were performed using the stats package in R (https://www.R-project.org/, accessed on 3 January 2026).

### 2.5. WGCNA Analysis

WGCNA was performed on the DEGs using the WGCNA package (version 1.72-1) [17]. This network is a scale-free, weighted gene network. To meet the construction criteria, we selected the minimum power value that achieved a scale-free topology fit index of 0.8 as the soft-thresholding parameter for the analysis. After evaluating the power values from 1 to 30, the recommended value of 8 was ultimately chosen to build the gene co-expression network, which clustered all genes into several modules. To identify biologically significant gene modules, we calculated the Pearson correlation coefficients between module eigengenes and sample traits, including disease-resistant and disease-susceptible phenotypes. To reduce potential false positives caused by multiple comparisons, *p*-values were adjusted using the Benjamini–Hochberg false discovery rate (FDR) correction. The results are summarized in a module–trait relationship heatmap. Each module was visualized using Cytoscape (v3.10.4) to illustrate the gene interaction networks [33].

## 3. Results

### 3.1. Transcriptome Sequencing Analysis

**Figure 1:**
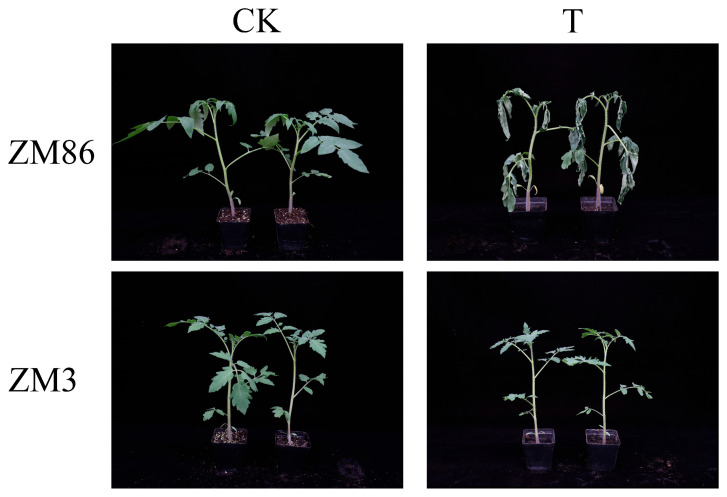
Phenotypes of tomato lines ‘ZM3’ and ‘ZM86’ at 4 days post-inoculation (dpi) with *R. solanacearum*.

In this study, transcriptome profiling was conducted on the root, stem, and leaf tissues of the strongly resistant tomato variety ‘ZM3’ and the susceptible tomato variety ‘ZM86’ at 0 and 4 days after inoculation with *R. solanacearum* (Figure 1). ‘ZM86’ exhibited pronounced wilting symptoms, whereas ‘ZM3’ exhibited no visible disease symptoms.

**Figure 2:**
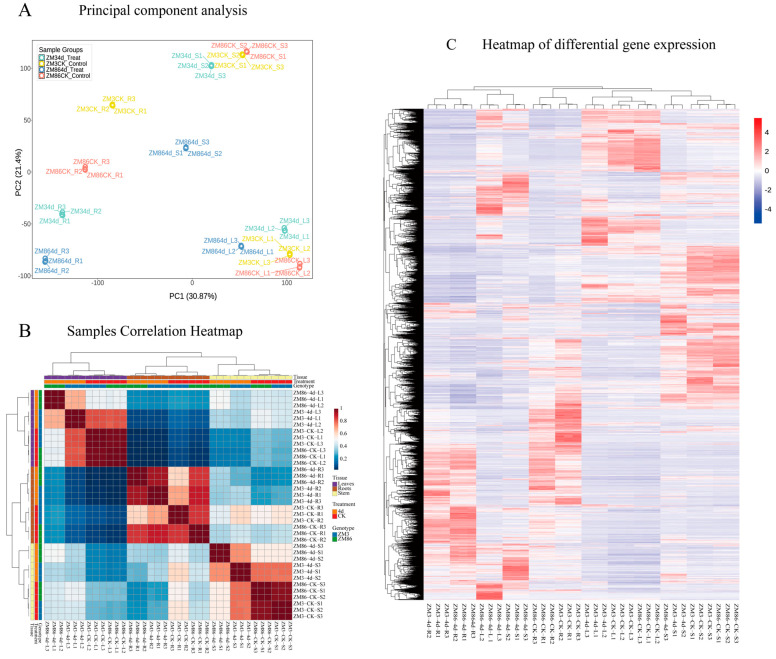
Sample correlation analysis of transcriptome data. (**A**) Principal component analysis (PCA) showing clustering of biological replicates. (**B**) Pearson correlation coefficients among transcriptome samples. (**C**) Heatmap of sample clustering analysis based on differentially expressed genes.

RNA sequencing generated 95.06 GB of high-quality clean reads, which were mapped to the tomato reference genome. The overall alignment rates ranged from 98.85% to 99.90%, with 97.07–99.27% of reads mapped to annotated gene regions (Table S1). PCA revealed tight clustering of biological replicates within each treatment group, indicating high reproducibility (Figure 2A). Hierarchical clustering of differentially revealed uniform expression patterns among replicates across all comparisons, collectively confirming the robustness and reliability of the transcriptome dataset for subsequent analyses (Figure 2C). Moreover, Pearson’s correlation analysis demonstrated exceptionally high concordance among biological replicates, with correlation coefficients exceeding 0.993 (Figure 2B). Collectively, these results confirmed the robustness, consistency, and reliability of the transcriptome dataset, supporting its suitability for downstream analyses. Furthermore, after inoculation treatment, genes in the three different tissues of ‘ZM3’ exhibited strong expression similarity following infection. In ‘ZM86’, the expression similarity was relatively weak in leaves but notably strong in roots. Moreover, post-infection, both ‘ZM3’ and ‘ZM86’ displayed a strong negative correlation between the expression patterns of leaf and root genes. This contrasting pattern may reflect tissue-specific defense strategies, where roots serve as the primary site of pathogen invasion while leaves undergo systemic transcriptional adjustments associated with stress signaling and resource reallocation.

### 3.2. DEGs, GO, and KEGG Enrichment Analysis

**Figure 3:**
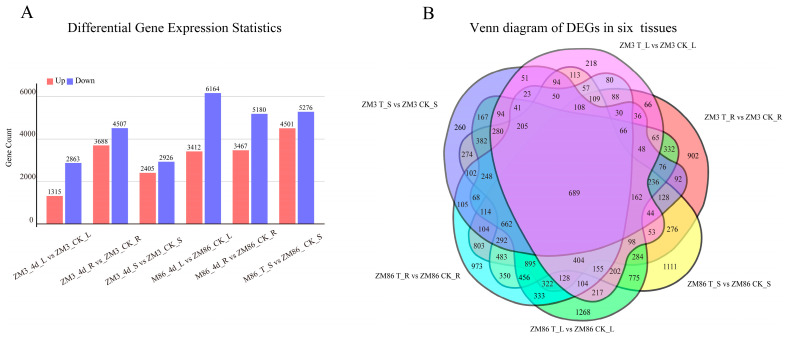
Analysis of DEGs. (**A**) Number of DEGs identified in roots, stems, and leaves of the susceptible line ‘ZM86’ and the resistant line ‘ZM3’ at 0 days post-inoculation (0 dpi, CK) and 4 days post-inoculation (4 dpi). (**B**) Venn diagrams showing the overlap of DEGs among three tissues in the two genotypes. Red represents DEGs in ZM3 roots before and after bacterial wilt infection treatment; yellow represents DEGs in ZM86 shoots before and after bacterial wilt infection treatment; green represents DEGs in ZM86 leaves before and after bacterial wilt infection treatment; cyan represents DEGs in ZM86 roots before and after bacterial wilt infection treatment; blue represents DEGs in ZM3 shoots before and after bacterial wilt infection treatment; purple represents DEGs in ZM3 leaves before and after bacterial wilt infection treatment. The numbers within the colored blocks indicate the count of DEGs in the corresponding subset or intersection.

Compared to the control group (CK), a substantial number of DEGs were identified in the root, stem, and leaf tissues of both the resistant cultivar ‘ZM3’ and the susceptible cultivar ‘ZM86’ following inoculation with *R. solanacearum*. Notably, the number of DEGs in all three tissues of the resistant cultivar ‘ZM3’ was consistently lower than that in the susceptible cultivar ‘ZM86’ (Figure 3A).

Specifically, in the resistant cultivar ‘ZM3’, 8195 (3688 upregulated and 4507 downregulated), 5331 (2405 upregulated and 2926 downregulated), and 4178 (1315 upregulated and 1863 downregulated) DEGs were identified in the roots, stems, and leaves, respectively. In the susceptible cultivar ‘ZM86’, 8647 (3467 upregulated and 5180 downregulated), 9777 (4501 upregulated and 5276 downregulated), and 9576 (3412 upregulated and 6164 downregulated) DEGs were detected in the roots, stems, and leaves, respectively (Figure 3A).

The DEG datasets from the six different comparison groups (representing the two genotypes across the three tissues) were analyzed using a Venn diagram (Figure 3B). This analysis revealed that 689 DEGs were common to all six genotype-tissue combinations. Within these datasets, common DEGs were identified in the same tissues between ‘ZM3’ and ‘ZM86’, with 803 in the roots, 274 in the stems, and 217 in the leaves. Additionally, the number of unique DEGs (i.e., genes exclusively differentially expressed in a single genotype-tissue combination) was determined. In ‘ZM3’, the roots, stems, and leaves contained 902, 1111, and 218 unique DEGs, respectively. In contrast, the roots, stems, and leaves of ‘ZM86’ contained 973, 260, and 1268 unique DEGs, respectively (Figure 3B).

**Figure 4:**
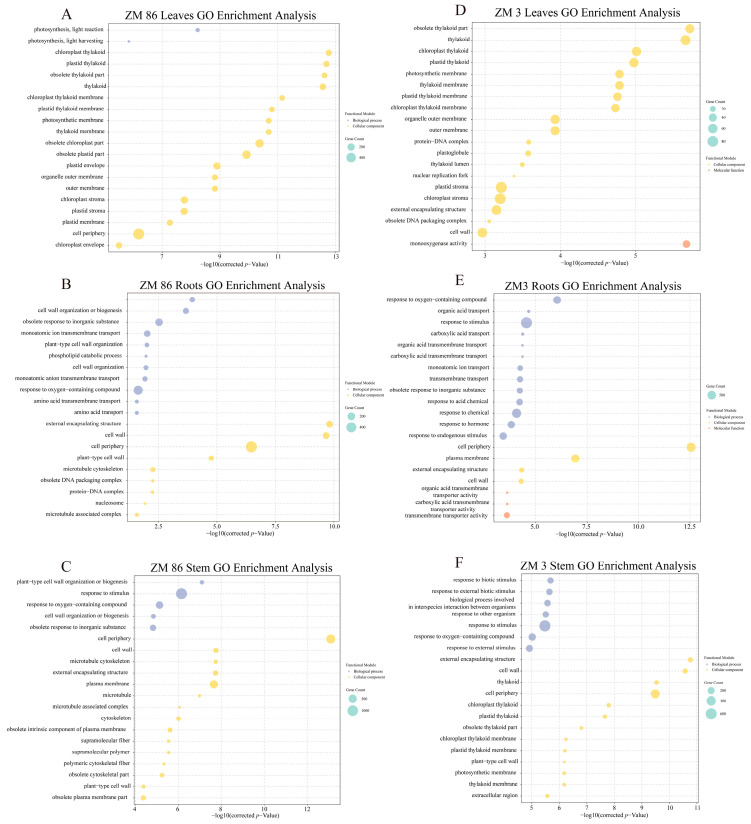
Gene Ontology (GO) Enrichment analysis of DEGs. Enriched GO terms derived from DEGs in leaves, roots, and stems of the susceptible line ‘ZM86’ (**A**–**C**) and the resistant line ‘ZM3’ (**D**–**F**) following *R. solanacearum* infection.

GO functional enrichment analysis was conducted on the DEGs from both ‘ZM3’ and ‘ZM86’ (Figure 4). The 20 most significantly enriched terms (padj < 0.05) were selected for visualization. Based on the enrichment results, the root tissues of ‘ZM3’ and ‘ZM86’ shared numerous common “GO terms”, including “cell periphery”, “cell wall”, and “organic acid transport”. In the “biological process category”, ‘ZM3’ was primarily enriched for terms such as “response to oxygen-containing compound”, “organic acid transport”, and “response to stimulus”, while ‘ZM86’ showed enrichment for “response to oxygen-containing compound”, “monoatomic ion transport”, and “obsolete cellular lipid catabolic process”. For “cellular components”, terms uniquely enriched in ‘ZM3’ included “membrane and plasma membrane”, whereas ‘ZM86’ exhibited unique enrichment for “obsolete DNA packaging complex” and “nucleosome”. In the “molecular function category”, ‘ZM3’ was dominated by “organic acid transmembrane transporter activity” and “carboxylic acid transmembrane transporter activity”, while ‘ZM86’ was characterized by “pectinesterase inhibitor activity” and “oxidoreductase activity”. In the stem tissues of both materials, common pathways included “external encapsulating structure”, “cell wall”, and “thylakoid”. ‘ZM3’ was mainly enriched for “obsolete thylakoid” part, “chloroplast thylakoid membrane”, and “plastid thylakoid membrane”, whereas ‘ZM86’ was enriched for “microtubule cytoskeleton”, “plant-type cell wall” organization or “biogenesis”, and “microtubule”. In the leaves of ‘ZM3’ and ‘ZM86’, common terms included “obsolete thylakoid” part, “thylakoid”, and “chloroplast thylakoid”. Unique pathways in ‘ZM3’ involved “brassinosteroid homeostasis”, “nuclear replication fork”, “membrane”, “cell periphery”, and “monooxygenase activity” (Figure 4).

**Figure 5:**
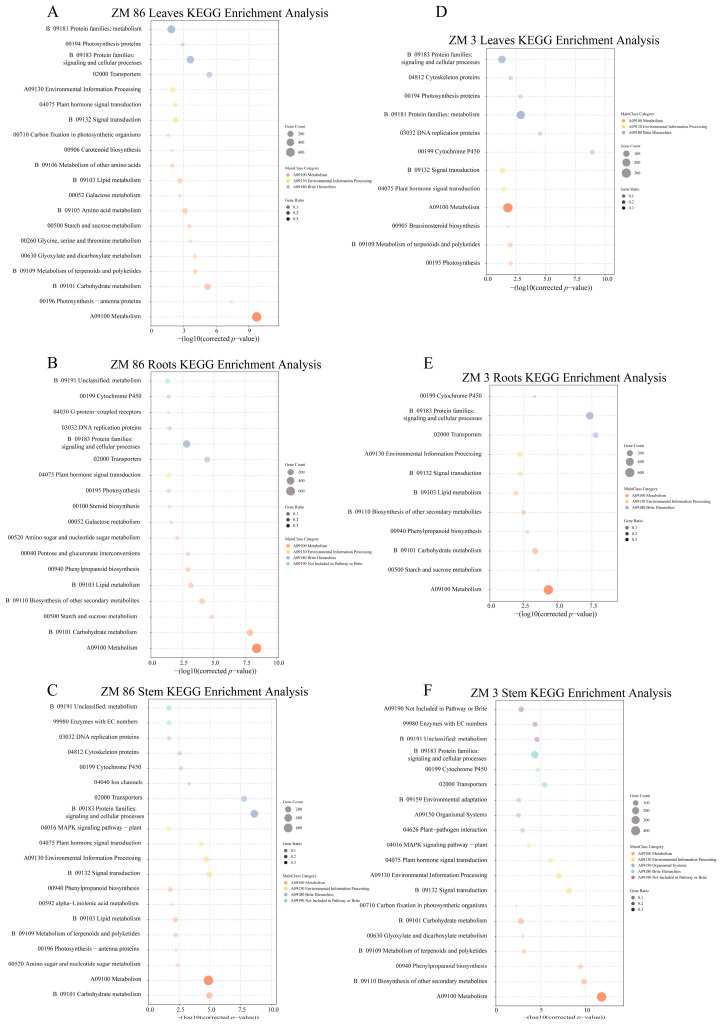
KEGG Enrichment analysis of DEGs. Enriched KEGG pathways derived from DEGs in leaves, roots, and stems of the susceptible line ‘ZM86’ (**A**–**C**) and the resistant line ‘ZM3’ (**D**–**F**). Dot size represents gene count, and color indicates gene ratio.

KEGG enrichment analysis was performed to predict the functions of the DEGs. Top 20 most significantly enriched pathways (based on padj). were analyzed (Figure 5). Many identical pathways were enriched in both the resistant material ‘ZM3’ and the susceptible material ‘ZM86’, with the majority of genes enriched in pathways such as “Cytochrome P450”, “Metabolism”, “Plant hormone signal transduction”, and “Signal transduction”. In contrast to ‘ZM3’, where genes were enriched in “Photosynthesis”, “Glycerophospholipid metabolism”, and “Ion channels”, the susceptible material ‘ZM86’ showed enrichment in distinct pathways including “Carbohydrate metabolism”, “Pentose and glucuronate interconversions”, and “Chaperones and folding catalysts”. In the root tissues of different genotypes, the top 20 enriched pathways were largely overlapping, such as “Transporters”, “Protein families: signaling and cellular processes”, “Metabolism”, “Starch and sucrose metabolism”, and “Carbohydrate metabolism”. In stem tissues, several key pathways common to both genotypes were identified, primarily pointing to “Protein families: signaling and cellular processes” and “Metabolism”. Moreover, the pathways of “Metabolism”, “Biosynthesis of other secondary metabolites”, “Phenylpropanoid biosynthesis”, and “Signal transduction” exhibited similar enrichment levels in the DEGs of both materials (Figure 5).

Pathways including “Cytochrome P450”, “Metabolism”, “Plant hormone signal transduction”, “Signal transduction”, “Protein families: signaling and cellular processes”, “Transporters”, and “Environmental Information Processing” were present across all enriched pathways. This indicates that signal transduction and metabolic synthesis pathways responded strongly to infection by *R. solanacearum*.

### 3.3. Transcriptomic Analysis of Genes Involved in JA, SA, BR, and ET Signaling Pathways

**Figure 6:**
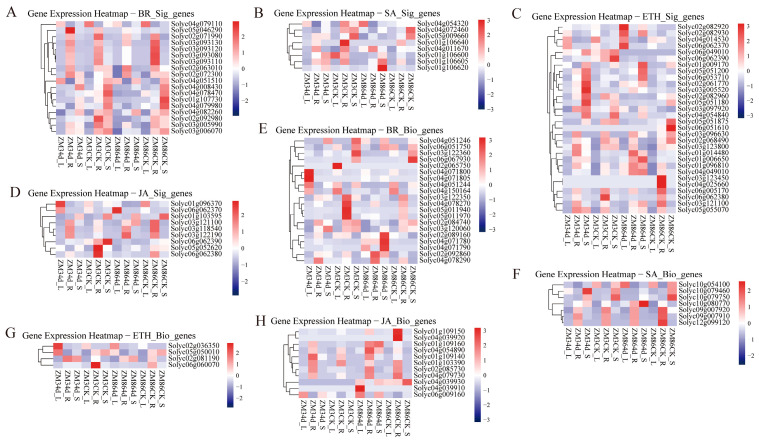
Heatmap of phytohormone biosynthesis- and signaling-related gene expression. Heatmaps illustrate the expression profiles of genes involved in brassinosteroid (BR), salicylic acid (SA), ethylene (ETH), and jasmonic acid (JA) pathways. Panels show genes associated with (**A**) BR signaling, (**B**) SA signaling, (**C**) ETH signaling, (**D**) JA signaling, (**E**) BR biosynthesis, (**F**) SA biosynthesis, (**G**) ETH biosynthesis, and (**H**) JA biosynthesis. Gene expression values were log-transformed and scaled by row. Color gradients from red to blue indicate high to low relative expression levels.

Pathway analysis revealed that signaling-related pathways, including “plant hormone signal transduction” and “signal transduction”, were enriched among the DEGs. Concurrently, the “Cytochrome P450 pathway”, potentially associated with “brassinolide (BR) biosynthesis”, was also enriched. Therefore, we selected the disease resistance-related hormones—BR, JA, ET and SA (involved in disease resistance signaling)—to generate the expression profiles of the pathway genes. A heatmap was constructed using DEGs to visualize the genes involved in the metabolism and signal transduction of the four hormones (Figure 6). For brassinolide, although the DEGs enrichment results did not directly indicate BR biosynthesis, pathways related to it, such as “Cytochrome P450” and “DWARF” (for subsequent analysis), were enriched. The heatmap shows that within the signal transduction pathway (from *Solyc05g046290* to *Solyc02g06310*; top to bottom in Figure 6A), these genes were specifically expressed in the roots. Notably, the expression of *Solyc05g046290* changed substantially in the roots of the resistant cultivar ‘ZM3’ following infection with *R. solanacearum*. A decreasing trend in gene expression was observed in the biosynthesis pathway (*Solyc03g1223350* and *Solyc02g084740*) after infection with *R. solanacearum* in both the resistant and susceptible cultivars.

Regarding ethylene signaling-related genes (*Solyc05g051200* to *Solyc04g054840*), pronounced upregulation was detected in ‘ZM3’ after *R. solanacearum* infection. Genes such as ethylene-responsive transcription factor 1a (*Solyc03g005520*) were significantly upregulated. In contrast, only three analogous genes in ‘ZM86’ exhibited relatively distinct upregulation. This pattern may also reflect potential crosstalk between ethylene and brassinosteroid signaling pathways. Previous studies have suggested that ethylene-mediated defense responses can interact antagonistically or coordinately with brassinosteroid signaling, thereby fine-tuning immune responses during pathogen challenge. The biosynthesis and signal transduction pathways of salicylic acid (SA), particularly the biosynthetic pathway, have only recently been clarified. Moreover, compared to Arabidopsis thaliana, homologs of SA biosynthesis genes in tomato have not been extensively reported. As the two primary SA biosynthesis pathways have been largely elucidated only in recent years, we examined the annotated genes relevant to SA biosynthesis in tomato. Excluding the genes that remained unannotated, *Solyc01g109140*, *Solyc01g103390*, and *Solyc06g09160* were upregulated in the roots, stems, and leaves after infection with *R. solanacearum*. After *R. solanacearum* infection, *Solyc03g121100* and *Solyc03g118540* were increased in the roots of ‘ZM3’ and decreased in the leaves of ‘ZM86’, respectively. *Solyc01g103595* exhibited reduced expression in stems across plant genotypes. It should be noted that additional components of the SA biosynthetic pathway may remain incompletely annotated in the tomato genome, and future studies incorporating homology-based searches and updated genome annotations may help further refine the identification of SA-related genes.

### 3.4. Key Gene Modules for R. solanacearum Infection Screened by WGCNA

**Figure 7:**
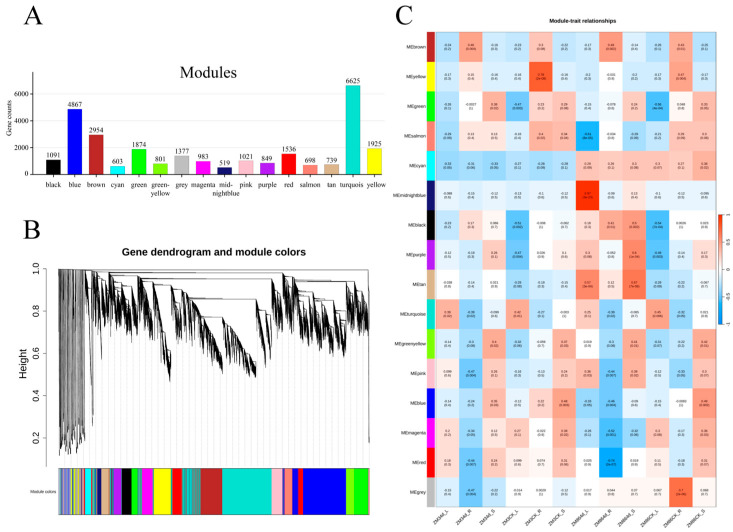
Key gene modules and Hub genes for *R. solanacearum* infection screened by WGCNA. (**A**) Number of genes in each module. (**B**) Cluster dendrogram of DEGs based on WGCNA, Each color represents a module in (**A**). (**C**) Correlation significance analysis between gene modules and traits. Red to blue represents relative Pearson levels ranging from 1 to −1.

We performed WGCNA based on the expression levels (TPM) of DEGs to further investigate the relationship between differential gene expression and traits in response to *R. solanacearum* infection across different tissues of the resistant cultivar ‘ZM3’ and susceptible cultivar ‘ZM86’. The resulting gene clusters were organized into a hierarchical tree, which successfully identified 16 distinct co-expression modules with stable expression patterns (Figure 7A). Notably, these modules exhibited considerable variation in size, with gene counts ranging from 519 to 6625 (Figure 7B). Correlation coefficients between sample trait characteristics were analyzed and visualized using a module-trait relationship heatmap to identify the key modules associated with *R. solanacearum* infection (Figure 7C).

The results demonstrated that prior to infection, genes in the ME yellow module (MTR = 0.782, *p* = 1.756 × 10^−8^) were highly correlated with the root tissue of ‘ZM3’. Following infection, the ME midnightblue module (MTR = 0.973, *p* = 2.629 × 10^−23^) and the ME tan module (MTR = 0.567, *p* = 3.120 × 10^−5^) showed strong correlations with the leaf tissue of ‘ZM86’. Additionally, the ME tan module was highly correlated with the stem tissue of ‘ZM86’ (MTR = 0.673, *p* = 6.930 × 10^−6^). Specifically, the ME red module (MTR = −0.742, *p* = 2.281 × 10^−7^) and the ME salmon module (MTR = −0.609, *p* = 8.067 × 10^−5^) exhibited significant negative correlations with the root and leaf tissues of ‘ZM86’ after infection, respectively.

### 3.5. Identification, Functional Annotation, and RT-qPCR Validation of Hub Genes

**Figure 8:**
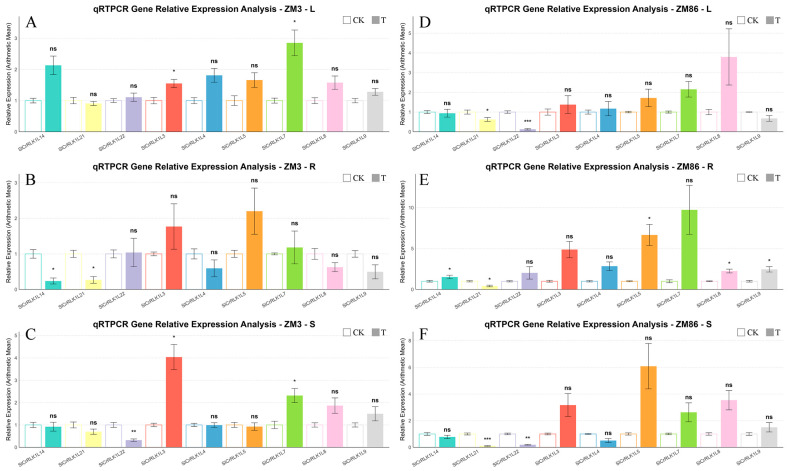
Validation of RNA-seq results by qRT–PCR. Relative expression levels of SlCrRLK1L genes were quantified by qRT–PCR and normalized to *SlActin* (*Solyc03g078400*). Expression levels were compared between the control group (CK, 0 dpi) and the treatment group (T, 4 dpi). Analyses were conducted in leaves, roots, and stems of the resistant cultivar ‘ZM3’ (**A**–**C**) and the susceptible cultivar ‘ZM86’ (**D**–**F**). Data represent the mean ± SD of three biological replicates. Statistical significance is indicated as ns (not significant), * *p* < 0.05, ** *p* < 0.01, *** *p* < 0.001.

**Figure 9:**
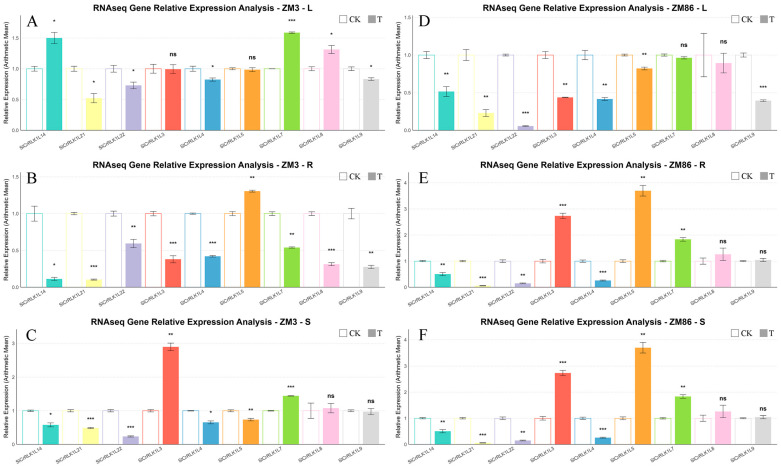
TPM-normalized expression profiles of the *SlCrRLK1L* gene family. Relative expression levels of *SlCrRLK1L* genes were estimated based on transcript per million (TPM) values. Open bars indicate the control group (CK, 0 dpi), and filled bars indicate the infected group (4 dpi). Expression patterns are shown for leaves, roots, and stems of the resistant line ‘ZM3’ (**A**–**C**) and the susceptible line ‘ZM86’ (**D**–**F**). Statistical significance is denoted as ns (not significant), *: *p* < 0.05, **: *p* < 0.01, ***: *p* < 0.001.

**Figure 10:**
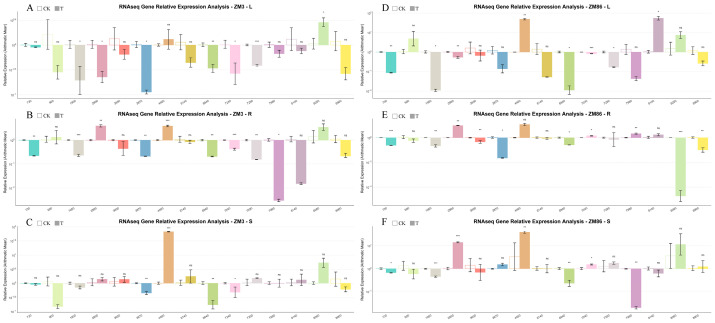
Relative Expression of picked module genes (**A**–**C**) Relative expression of selected module genes in leaves (L), roots (R), and stems (S) of ‘ZM3’; (**D**–**F**) relative expression of selected module genes in leaves (L), roots (R), and stems (S) of ‘ZM86’. Expression values were log-transformed and plotted on a logarithmic scale using R. Hollow bars represent the control group (CK, 0 DPI), while solid bars indicate the treated group (T, 4 DPI); each color corresponds to a single gene (see Table S3 for details). Statistical significance was determined using Student’s *t*-test, or Welch’s *t*-test when variances were unequal. Significance levels were calculated in R (version 4.4.2) and annotated manually. Significance levels are indicated as follows: ns, not significant; *p < 0.05; **p < 0.01; ***p < 0.001.

Representative genes from the previously reported tomato *SlCrRLK1L* gene family were selected for RT–qPCR analysis to validate the RNA-seq data. The expression patterns obtained by RT–qPCR were highly consistent with those from RNA-seq, confirming the reliability of the transcriptome analysis (Figure 8 and Figure 9). RT-qPCR was also employed to amplify some candidate hub genes (Figure 10).

**Figure 11:**
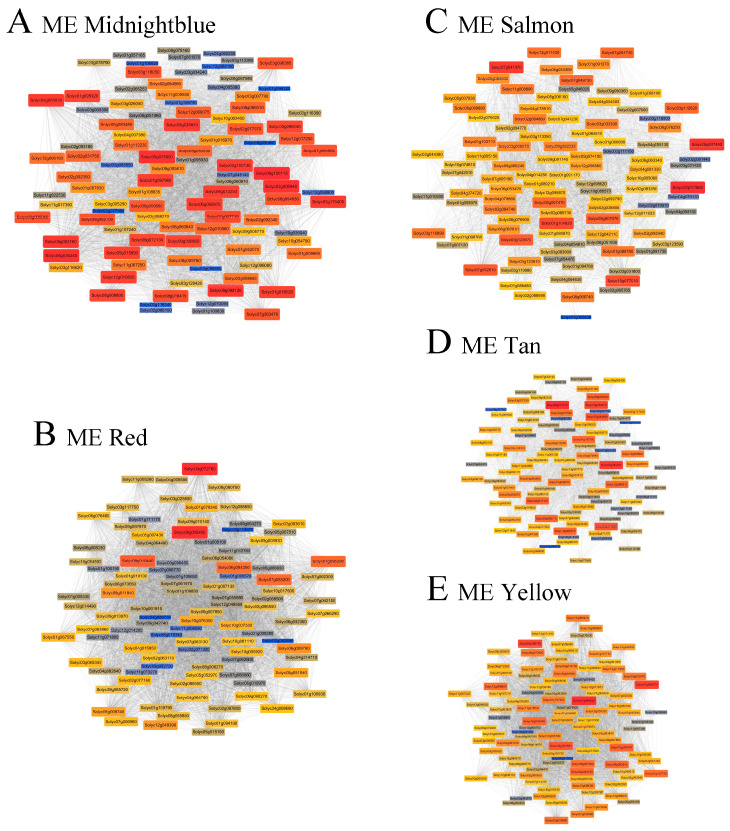
Co-expression networks of hub genes identified by WGCNA. Networks display the top 50 genes with the highest intramodular connectivity from five key modules: (**A**) ME midnightblue, (**B**) ME red, (**C**) ME salmon, (**D**) ME tan, and (**E**) ME yellow. Node color (red to blue) and node size (large to small) indicate relative gene connectivity within each module, with warmer colors and larger nodes representing higher connectivity. The top 10 genes for each module and their detailed information can be found in Supplementary Table S3.

Based on module–trait associations, five key modules (MEmidnight Blue, ME tan, ME red, ME salmon, and ME yellow) were selected for gene network construction and hub gene identification. Co-expression networks were visualized using the top 50 genes with the highest intramodular connectivity in each module (Figure 11). Genes lacking functional annotations (hypothetical proteins) were excluded from subsequent analyses (Table S2). Although several hub genes in the identified modules were annotated as hypothetical proteins and were therefore not included in the detailed functional discussion, these genes may still play important roles in the resistance network. Future studies incorporating protein domain prediction and functional annotation analyses may help reveal their potential biological roles in tomato defense responses.

In the positively correlated modules, the candidate hub genes included ACO (*Solyc04g007980*), SP3C (*Solyc03g026050*), a peroxisomal membrane 22 kDa protein (*Solyc01g107240*), and a metallothionein-like protein (*Solyc01g112230*), all of which are associated with hormone signaling, redox homeostasis, and stress adaptation. Notably, ACO was identified in both the ME midnightblue and ME tan modules, whereas ERF1 (*Solyc09g091950*) emerged as a key hub in ME yellow, indicating a prominent role of ethylene signaling in disease response regulation.

In the negatively correlated ME red module, hub genes were mainly enriched in immune signaling components, including RING/U-box superfamily protein (*Solyc02g082890*), MAPK9 (*Solyc04g080730*), receptor-like protein kinase (*Solyc07g006770*), and bHLH transcription factor (*Solyc08g078340*). Similarly, ME salmon contained several defense-related hubs, such as PAD4 (*Solyc02g067660*), 14-3-3 protein (*Solyc11g010200*), and SWEET sugar transporter (*Solyc04g064630*), suggesting coordinated regulation of immune signaling and metabolic reprogramming.

In the ME yellow module, additional hub genes included ERF1, a calcium-dependent protein kinase (*Solyc01g006840*), a dirigent protein (*Solyc10g008900*), and an acyltransferase-like protein (*Solyc11g067290*), indicating the integration of hormone signaling, calcium signaling, and secondary metabolism during resistance responses.

Collectively, the enrichment of transmembrane proteins, transporters, kinases, and transcription factors among the hub genes suggested that these modules occupied a central position in tomato defense networks. The contrasting distribution of immune-related hub genes between positively and negatively correlated modules further implies that resistance outcomes are shaped by the coordinated modulation of PTI/ETI-associated signaling pathways and pathogen-driven suppression mechanisms.

## 4. Discussion

We constructed a gene co-expression network using WGCNA based on transcriptomic data from different genotypes and tissues following infection. This analysis resolved distinct gene modules associated with host resistance, providing a systems-level view of tomato immune responses across the root, stem, and leaf tissues to identify the key genes and associated resistance pathways involved in tomato responses to bacterial wilt caused by *R. solanacearum*. Notably, several genes within the resistance-related modules exhibited expression patterns consistent with previously reported pathogen-responsive genes, including those implicated in interactions with bacterial pathogens such as *Pseudomonas syringae* [14], supporting the biological relevance and reliability of our dataset. Plant disease resistance is inherently complex and mediated by multilayered signaling networks that involve both pattern-triggered immunity (PTI) and effector-triggered immunity (ETI) [34]. In this context, network-based approaches such as WGCNA provide an effective framework for dissecting coordinated gene regulation and identifying the key hub genes underlying resistance traits. Compared with traditional QTL mapping, co-expression network analysis enables more efficient prioritization of candidate genes by integrating expression dynamics and functional connectivity, thereby facilitating the elucidation of the regulatory mechanisms governing tomato resistance to *R. solanacearum* [35].

Our GO enrichment analysis revealed significant enrichment of “peripheral cell” and “cell wall”, consistent with the infection strategy of *R. solanacearum*, which invades host tissues through root-associated cell wall interfaces [2]. This suggests that early defense responses are predominantly activated at plant–pathogen contact sites. In parallel, KEGG pathway analysis showed strong enrichment of “plant hormone signal transduction”, “signal transduction”, and “plant–pathogen interaction”, indicating that pathogen perception triggers extensive immune signaling cascades. A similar enrichment of defense-related metabolic and secondary metabolite biosynthesis pathways has been reported in resistant tomato genotypes, supporting the robustness of our findings [36]. Notably, recent studies have identified LysM-type receptor kinases, such as *SlLYK4*, as key components in recognizing *R. solanacearum* exopolysaccharides on the cell surface and mediating immune activation via differential phosphorylation [37]. Collectively, these results highlight the complexity and coordination of cell surface recognition, hormone signaling, and downstream defense responses in tomato resistance to bacterial wilt.

At the transcriptome level, we systematically compared genes associated with four major phytohormone signaling pathways (ethylene, salicylic acid, jasmonic acid, and brassinosteroids) across genotypes, tissues, and infection stages. KEGG enrichment analysis revealed significant involvement of the “plant hormone signal transduction” and “brassinolide biosynthesis” pathways during *R. solanacearum* infection, indicating a central role for hormone-mediated regulation in tomato defense. Based on these enrichment results and the well-established roles of ET, SA, and JA in plant–pathogen interactions [38], we further examined the expression dynamics and potential functions of genes associated with these defense-related hormones. The analysis showed that the ET-, SA-, and JA-related genes exhibited coordinated and genotype-dependent transcriptional responses following infection, supporting their roles as core components of tomato immune regulation. In contrast, BR-related genes, including BZR1, DWF4, and BAK1, were preferentially upregulated in resistant genotypes and closely followed disease progression, suggesting that BR signaling may participate in resistance-associated transcriptional reprogramming and potentially interact with MAPK-mediated defence pathways [39,40]. Collectively, these findings indicate that ET, SA, and JA constitute the central hormonal framework underlying tomato resistance to *R. solanacearum*, whereas BR may function as a modulatory signal that could contribute to immune regulation through pathway integration.

Among these pathways, ethylene signaling has emerged as the central regulatory axis. Consistent with previous VIGS studies in resistant tomato lines [13], ethylene biosynthesis genes such as *ACO* family members displayed stronger and more sustained induction in the resistant genotype ‘ZM3’ than in the susceptible ‘ZM86’. Notably, ERF transcription factors, which integrate ET and JA signals, showed genotype- and tissue-specific expression patterns, with ERF1 retained in resistant roots, but broadly repressed elsewhere following infection. This suggests that *R. solanacearum* actively suppresses ethylene-responsive transcription, whereas resistant plants maintain localized ET signaling competence, potentially mitigating xylem dysfunction and the severity of wilting. Collectively, these findings highlight the central role of hormone-mediated signaling networks in shaping tomato resistance to *R. solanacearum*. However, defence activation is not solely governed by immune signaling pathways but is also closely associated with broader metabolic adjustments required for stress adaptation. Beyond immune signaling pathways, the observed transcriptional shifts may also reflect broader metabolic reprogramming associated with plant stress adaptation. Activation of defence responses is known to impose substantial energetic costs, often resulting in trade-offs between growth and immunity [41]. In this context, resistant genotypes may maintain a more coordinated transcriptional adjustment that balances defence activation with metabolic homeostasis, whereas susceptible plants may experience stronger transcriptional disruption following infection. An alternative interpretation is that the observed gene expression patterns reflect differences in resource allocation strategies during pathogen challenge, where resistant plants preserve key regulatory and metabolic functions to sustain defence responses. Such energy trade-offs and metabolic adjustments are increasingly recognized as critical components of plant immune resilience and may contribute to the differential resistance observed in tomato against *R. solanacearum*.

Network analysis further revealed that key components of PTI/ETI signaling, including MAPKs, receptor-like kinases, calcium-dependent protein kinases, and bHLH transcription factors, were predominantly downregulated following infection, particularly in susceptible tissues. This widespread repression suggests that R. solanacearum interferes with host immune signal transduction at multiple regulatory nodes. Collectively, our results indicate that tomato resistance to bacterial wilt may depend on the plant’s ability to preserve critical hormone-mediated and PTI/ETI signaling modules, especially the ethylene-centered network, against pathogen-driven transcriptional suppression. These findings highlight the importance of regulatory resilience rather than simple pathway activation in shaping disease outcomes.

Collectively, the differential susceptibility of tomato to bacterial wilt is governed by the coordinated regulation of phytohormone signaling and associated defense pathways, reflecting a complex immune network shaped by synergistic and antagonistic hormone interactions [42]. As Ralstonia solanacearum invades host roots and establishes within xylem vessels [43], tomato activates both structural and chemical defense programs. Although dirigent family proteins associated with lignin deposition were transcriptionally induced following infection, their comparable expression across genotypes and time points suggests that lignification alone is unlikely to determine resistance under the root-wound inoculation conditions used in this study. In contrast, several genes involved in antimicrobial metabolite biosynthesis have emerged as potentially important contributors to defense mechanisms. Notably, the HXXXD-type acyltransferase Solyc11g067290 belongs to the BAHD enzyme family, which catalyzes acyl transfer between hydroxycinnamoyl-CoA derivatives and aliphatic alcohols, producing alkyl hydroxycinnamates with documented antimicrobial activity against soil-borne bacteria, including Pseudomonas fluorescens [44,45]. In parallel, gibberellin-regulated protein 3 (*Solyc02g083870*) play a dual role, responding to hormonal cues while exhibiting structural features characteristic of antimicrobial peptides [46]. Collectively, these genes highlight a chemical defense layer that may restrict bacterial proliferation during vascular colonization. Notably, the repression of these antimicrobial-related genes in both the resistant and susceptible lines suggests that *R. solanacearum* actively suppresses host chemical defenses, mirroring its interference in hormone-responsive signaling pathways. Integrating transcriptomic profiling with WGCNA further revealed that tomato resistance was orchestrated through coordinated jasmonate, salicylate, and brassinosteroid signaling, with ethylene acting as a central regulatory hub. Simultaneously, the pathogen appears to deploy a dual suppression strategy targeting both PTI/ETI signaling nodes and antimicrobial metabolic pathways, thereby weakening host immunity and facilitating disease progression. These findings emphasize that resistance to bacterial wilt depends not on isolated defense components but on the plant’s capacity to sustain interconnected hormonal and chemical defense networks against pathogen-driven transcriptional repression, providing a conceptual framework for prioritizing resistance-associated genes and developing multi-pathway breeding strategies. However, the present study focused on two infection time points (0 and 4 dpi), which primarily capture the transcriptional landscape associated with disease establishment rather than the earliest immune signaling events following pathogen perception. In addition, functional validation of the candidate genes identified through transcriptomic and network analyses was beyond the scope of this work. Future studies integrating finer temporal resolution and targeted functional assays will be essential to further elucidate the regulatory mechanisms underlying tomato resistance to *R. solanacearum*.

## 5. Conclusions

In this study, we performed a comparative transcriptome analysis of two tomato inbred lines, the highly resistant ‘ZM3’ and the susceptible ‘ZM86’ to elucidate key regulatory factors underlying resistance to bacterial wilt caused by R. solanacearum. Our results demonstrate that plant hormone signal transduction, plant–pathogen interaction, and MAPK signaling pathways play important roles in tomato defense responses following infection. Further expression profiling suggests that the coordinated regulation of ethylene-, jasmonic acid-, and salicylic acid-associated genes constitutes a central hormonal framework of resistance. In contrast, brassinosteroid-related genes exhibit genotype-dependent activation and may act as modulators through pathway crosstalk. Among the resistance-associated candidates, the BAHD family HXXXD-type acyltransferase *Solyc11g067290* and gibberellin-regulated protein 3 (*Solyc02g083870*) may contribute to chemical defense during xylem colonization, whereas the ethylene-responsive transcription factor ERF1 appears to function as a key regulatory node integrating hormone signaling pathways. Moreover, WGCNA revealed resistance-associated co-expression modules enriched in immune signaling components, highlighting coordinated regulation between PTI/ETI signaling and hormone-mediated defense networks. Collectively, these findings provide new insights into the molecular basis of tomato resistance to *R. solanacearum* and identify candidate genes and regulatory pathways that may serve as valuable resources for future functional studies and potential targets for breeding bacterial wilt-resistant tomato varieties.

## Data Availability

The original contributions presented in this study are included in the article/Supplementary Materials. Further inquiries can be directed to the corresponding author.

## Supplementary Materials

The following supporting information can be downloaded at: https://www.mdpi.com/article/10.3390/biology15060509/s1, Table S1: Summary of raw transcriptome sequencing data for all samples; Table S2: Primer sequences used for RT-qPCR analysis of *SlCrRLK1L* genes; Table S3: Functional annotation of the top 10 genes with the highest connectivity in each of the five WGCNA modules.
